# Origin, transmission, diagnosis and management of coronavirus disease 2019 (COVID-19)

**DOI:** 10.1136/postgradmedj-2020-138234

**Published:** 2020-06-20

**Authors:** Srikanth Umakanthan, Pradeep Sahu, Anu V Ranade, Maryann M Bukelo, Joseph Sushil Rao, Lucas Faria Abrahao-Machado, Samarika Dahal, Hari Kumar, Dhananjaya KV

**Affiliations:** Paraclinical Sciences, The University of the West Indies at Saint Augustine Faculty of Medical Sciences, Saint Augustine, Trinidad and Tobago; Centre for Medical Sciences Education, The University of the West Indies at Saint Augustine, Saint Augustine, Trinidad and Tobago; Basic Medical Sciences, University of Sharjah, Sharjah, United Arab Emirates; Department of Anatomical Pathology, North Central Regional Health Authority, Eric Williams Medical Sciences Complex, Mount Hope, Trinidad and Tobago; Department of Surgery, University of Minnesota, Minneapolis, Minnesota, USA; Department of Pathology, Bacchi Laboratory, Sao Paulo, Brazil; Department of Oral Pathology, Institute of Medicine, Kathmandu, Nepal; Department of International Public Health and Disease Surveillance, Midwest, Australia; Department of Radiology, Indiragandhi Institute of Child Health, Bangalore, India

**Keywords:** Pathology, Histopathology, Education and training (see medical education & training), Medical education and training, Surgery, Transplant surgery, Hepatobiliary surgery, Basic sciences, Pathology

## Abstract

Coronavirus has emerged as a global health threat due to its accelerated geographic spread over the last two decades. This article reviews the current state of knowledge concerning the origin, transmission, diagnosis and management of coronavirus disease 2019 (COVID-19). Historically, it has caused two pandemics: severe acute respiratory syndrome and Middle East respiratory syndrome followed by the present COVID-19 that emerged from China. The virus is believed to be acquired from zoonotic source and spreads through direct and contact transmission. The symptomatic phase manifests with fever, cough and myalgia to severe respiratory failure. The diagnosis is confirmed using reverse transcriptase PCR. Management of COVID-19 is mainly by supportive therapy along with mechanical ventilation in severe cases. Preventive strategies form the major role in reducing the public spread of virus along with successful disease isolation and community containment. Development of a vaccine to eliminate the virus from the host still remains an ongoing challenge.

## Introduction

Coronavirus (CoV) is derived from the word ‘corona’ meaning ‘crown’ in Latin.^1^ It causes a range of human respiratory tract infections varying from mild cold to severe respiratory distress syndrome.^2^ The present novel CoV disease also called as severe acute respiratory syndrome (SARS)-CoV-2 and coronavirus disease 2019 (COVID-19) is an emerging global health threat.^3^ The COVID-19 epidemic started from Wuhan city of China towards the end of December 2019 and since then spread rapidly to Thailand, Japan, South Korea, Singapore and Iran in the initial months.^4–6^ This was followed by wide viral dissemination around the world including Spain, Italy, USA, UAE and the UK.^7^ The WHO declared the COVID-19 outbreak as a pandemic.^8^ As of 6 May 2020, outbreaks and sporadic human infections have resulted in 3 732 046 confirmed cases and 261 517 deaths.^7^

The CoV has posed frequent challenges during its course ranging from virus isolation, detection, prevention to vaccine development.^9^ CoV belongs to the order Nidovirales and has the largest RNA genome.^10^ It is known to be acquired from a zoonotic source and typically spreads through contact and droplet transmission. The infected person presents with non-specific clinical features requiring virological detection and confirmation by molecular techniques.^11–13^ This article aims to give a detailed insight into the evolution, transmission and diagnosis of COVID-19. We further discuss the challenges encountered in the management of patients with COVID-19 and the current limitations in the investigational vaccine. Due to the rapidly evolving nature of COVID-19, the readers are requested to update themselves with the nature of change with this particular type of CoV.

## ORIGIN

### Historic perspective

CoV was discovered during the 1960s. The Coronavirus Study Group under the International Committee on Taxonomy of Viruses used the principle of comparative genomics to further assess and partition the replicative proteins in open reading frames to identify the factors that differentiate CoV at different cluster ranks.^14  15^ CoV is associated with illness of varied intensity. The most severe type resulting in large-scale pandemics in the past are the SARS (in 2002–2003) and Middle East respiratory syndrome (MERS) (in 2012).^16  17^

### Aetiology

CoV are RNA viruses of the subfamily Coronavirinae. They belong to the family Coronaviridae and the order Nidovirales (nido Latin for ‘nest’). The order Nidovirales is composed of Coronaviridae, Arteriviridae, Mesovirididae and Roniviridae families.^10  18^ The characteristic features of Nidovirales are as follows: they (1) contain very large genomes for RNA viruses, (2) are highly replicative due to conserved genomic organisation, (3) exhibit several unique enzymatic activities and (4) have extensive ribosomal frameshifting due to the expression of numerous non-structural genes. The Coronaviridae family have two subfamilies: Coronavirinae and Torovirinae. The subfamily Coronavirinae consist of alpha CoV, beta CoV, gamma CoV and delta CoV based on genomic structure.^19^

### Viral structure

The CoV are enveloped positive single-stranded RNA viruses having the largest known viral RNA genomes of 8.4–12 kDa in size.^20^ The viral genomes are made up of 5ʹ and 3ʹ terminal. The 5ʹ terminal constitutes a major part of the genome and contains open reading frames, which encodes proteins responsible for viral replication. The 3ʹ terminal contains the five structural proteins, namely the spike protein (S), membrane protein (M), nucleocapsid protein (N), envelope protein (E) and the haemagglutinin-esterase (HE) protein.^21  22^ The S protein mediates an attachment and fusion between the virus and host cell membrane and also between the infected and adjacent uninfected cells. They are the major inducers for neutralising antibodies in a vaccine. The N protein forms RNA complexes that aid in virus transcription and assembly. The M protein is the most abundant structural protein and also defines the viral envelope shape. The E protein is the most enigmatic and the smallest of the major structural protein, which is highly expressed within the infected cell during viral replication cycle. The HE protein is responsible for receptor binding and host specificity.^20  23^

### SARS and MERS

SARS was first recognised in Guangdong province, China, in November 2002. It advanced among 30 countries, infecting 79 000 people by 2003 with a fatality of 9.5%. SARS-CoV was traced and isolated from Himalayan palm civets found in a livestock market in Guangdong, China.^16  24^ The zoonotic origin of SARS was also discovered in racoon dogs, ferret badgers and in humans working at the same market. These market animals were therefore intermediate hosts that increased the transmission of virus to humans.^15  24^

Thereon, in 2012, Jeddah, Saudi Arabia, a patient presented with respiratory illness consistent with pneumonia along with features of renal failure.^25^ The patient’s sputum analysis was done by reverse transciptase (RT-PCR) using pan-CoV primers revealing the viral RNA to be MERS-CoV.^26^ As of July 2013, 91 patients were infected with MERS-CoV and had a high fatality rate of 34%. Bats and Arabian dromedary camels were identified as potential hosts for MERS-CoV. Intermediate host reservoir species were also seen in goats, sheep and cows.^27  28^

### Novel CoV

In view of taxonomical classification, SARS-CoV-2 (COVID-19) is one among many other viruses in the species, SARS-related CoV. However, SARS-CoV and SARS-CoV-2 vary in terms of disease spectrum, modes of transmission and also diagnostic methods.^9  28^ The recent report on a cluster cases having respiratory illness in Wuhan, Central China, was followed by a global spread of the disease in a very short duration of time. The samples (oral and anal swabs, blood and broncho-alveolar fluid lavage) from patients admitted to the intensive care unit of Wuhan Jinyintan Hospital were sent to Wuhan Institute of Virology. Pan-CoV PCR primers were used and these samples were positive for CoV.^1–3  29^ This was followed by metagenomics analysis and genomic sequencing study. The results revealed that this virus was identical (79.6%) to the genetic sequence of SARS-CoVBJ01 leading the WHO to call it novel CoV-2019 (2019-nCoV).^30  31^

## Transmission and Pathogenesis

### Zoonosis

CoVs are widespread among birds and mammals with cements bats forming the major evolutionary reservoir and ecological drivers of CoV diversity.^32^ CoV causes a large variety of diseases in pigs, cows, chicken, dogs and cats. The major diseases caused by CoVs in animals are transmissible gastroenteritis virus, porcine epidemic diarrhoea virus, porcine hemagglutinating encephalomyelitis virus and murine hepatitis virus. In humans, alpha and beta CoV have caused a variety of illness ranging from mild-self-limiting respiratory infections (HCoV-229E, HCoV-NL63, HCoV-OC43, HCoV-HKU1) to severe acute respiratory distress syndrome (ARDS).^16  33–35^

Initial cases reported in Wuhan, China, are considered to be an acquired infection from a zoonotic source from Huanan wholesale seafood market which sold poultry, snake, bats and other farm animals.^36  37^ To isolate the possible virus reservoir, a comprehensive genetic sequence analysis was undertaken among different animal species.^9  15^ The results suggested that 2019-nCov is a recombinant virus between the bat CoV and an unknown origin CoV. A study revealed, based on relative synonymous codon usage (RSCU) on variety of animal species showed that bats are the most probable wildlife reservoir of 2019-nCov.^10^ This homologous recombination has proved previously in classical swine fever virus, hepatitis B virus, hepatitis C virus, HIV and dengue virus.^38^

### Modes of spread

**Figure 1:**
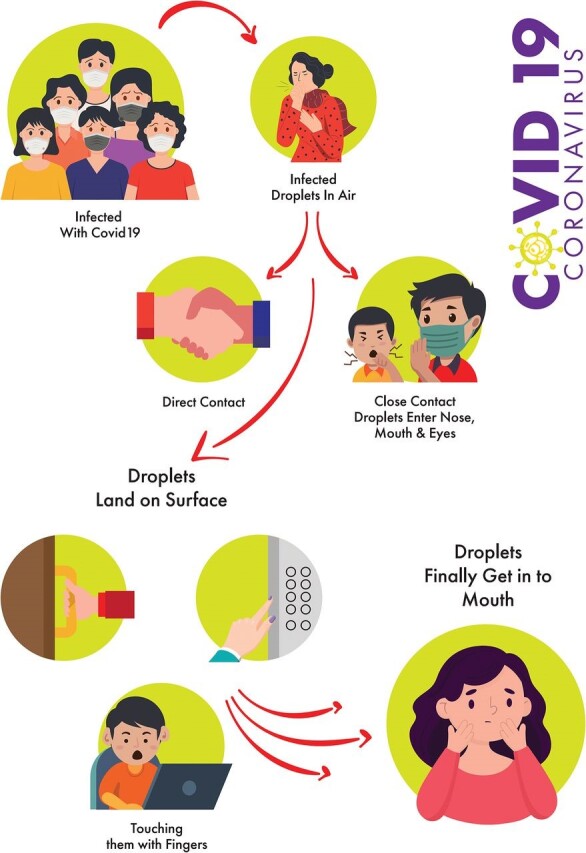
Modes of transmission.

Human-to-human transmission occurs through common routes such as direct transmission, contact transmission and airborne transmissions through aerosols and during medical procedures (figure 1). Cough, sneeze, droplet inhalation, contact with oral, nasal and eye mucous membranes are the common modes of spread. Viral shedding occurs from respiratory tract, saliva, faeces and urine resulting in other sources of virus spread.^37  39  40^ The viral load is higher and of longer duration in patients with severe COVID-19.^41^ Spread of COVID-19 from patients to health workers and flight attenders who were in close contact with the infected patients are also reported.^42^

### Virus–host interaction

Extensive structural analyses revealed atomic-level interactions between the CoV and the host. Cross-species and human-to-human transmission of COVID-19 is mainly dependent on spike protein receptor-binding domain and its host receptor ACE2.^23  43^ High expression of ACE2 was identified in lung (type II alveolar cells), oesophagus, ileum, colon, kidney (proximal convoluted tubules), myocardium, bladder (urothelial cells) and also recently the oral mucosa. ACE2 receptors provide entry of the virus into the host cells and also subsequent viral replication. The main factors involved in viral pathogenesis of 2019-nCov are spike 1 subunit protein, priming by transmembrane protease serine-2 (essential for entry and viral replication), ACE2 receptor–2019-nCov interaction and downregulation of ACE2 protein. These factors contribute to atrophy, fibrosis, inflammation and vasoconstriction resulting in host tissue injury.^43–45^

## Clinical Presentation and Diagnosis

### Demographics

Based on numerous studies published, the median age was 56 years (range 55–65 years) and males were predominately affected due to high ACE2 concentrations in them. The median onset of illness was 8 days (range 5–13 days).^46  47^ Due to limited comorbid data availability, it is important to correlate with previously proven susceptible factors to SARS and MERS-CoV infection, which includes smoking, hypertension, diabetes, cardiovascular disease and/or chronic illness.^16  24  25^ Based on the National Health Institute analysis in Italy, the average mortality age for patients suffering from COVID-19 was 81 years.^48^ In China, the case fatality rate (CFR) increased with age and showed CFR of 18% for patients above 80 years.^49^ This striking target to the elderly population is attributed to underlying chronic disorders and declined immune function. Declined immune function has been linked to cytokine storm syndrome (elevated circulating inflammatory cytokines) and hyper-inflammation syndrome. These syndromes are triggered by viral infections and are also predictors of fatality in patients with COVID-19.^50  51^ Children are less affected due to higher antibodies, lower prior exposure to the virus and relatively low levels of inflammatory cytokines in their systems.

### Signs and symptoms

Clinical features varied from mild illness to severe or fatal illness. The most common symptoms of COVID-19 were non-specific and mainly included fever, cough and myalgia. Other minor symptoms were sore throat, headache, chills, nausea or vomiting, diarrhoea, ageusia and conjunctival congestion. The COVID-19 was clinically classified into mild to moderate disease (non-pneumonia and pneumonia), severe disease (dyspnoea, respiratory frequency over 30/min, oxygen saturation less than 93%, PaO_2_/FiO_2_ ratio less than 300 and/or lung infiltrates more than 50% of the lung field within 24–48 hours) and critical (respiratory failure, septic shock and/or multi-organ dysfunction/failure).^12  52^ Many of the elderly patients who had severe illness had evidence of chronic underlying illness such as cardiovascular disease, lung disease, kidney disease or malignant tumours.^53^

### Laboratory evaluation and confirmation

Laboratory findings most consistent with COVID-19 were lymphocytopenia, elevated C reactive protein and elevated erythrocyte sedimentation rate. Lymphocytopenia is due to necrosis or apoptosis of lymphocytes. The severity of lymphocytopenia reflects the severity of COVID-19.^54–56^ Procalcitonin was commonly elevated and was associated with coinfection in majority of reported paediatric cases.^57  58^

Detection of COVID-19 is based on virological detection by RT-PCR using swabs (nasopharynx, oropharynx), sputum and faeces, chest radiograph and dynamic monitoring of inflammatory mediators (eg, cytokines).^59–61^ Faecal specimens detected for COVID-19 nucleic acid was equally accurate as of pharyngeal swab specimens.^60^ Patients with COVID-19 showed high blood levels of cytokines and chemokines such as interleukin (IL)-7, IL-8, IL-9, IL-10, granulocyte-colony stimulating factor, granulocyte-macrophage colony-stimulating factor ,tumour necrosis factor alpha and VEGFA.^50  62  63^

### Radiological findings

Most standard patterns observed on chest CT were ground-glass opacity, ill-defined margins, smooth or irregular interlobular septal thickening, air bronchogram, crazy-paving pattern and thickening of the adjacent pleura. Chest CT is considered to be a sensitive routine imaging tool for COVID-19.^64–66^

## Management

At the initial presentation of cluster infection, many cases were treated with antiviral therapy, antibacterial therapy and glucocorticoids. Observation forms the mainstay for those who have mild illness. Moderately ill patients with underlying chronic illness, immunocompromised conditions and pregnancy require hospitalisation.^67  68^

The anti-malarial drugs, hydroxychloroquine and chloroquine, showed promising results in early in vitro study.^69^ However, the most robust and recent study in patients with COVID-19 have not shown unequivocal evidence of benefits for the treatment with hydroxychloroquine or chloroquine.^70–72^ In fact, the largest analysis to date of the risks and benefits of treating COVID-19 patients with these anti-malarial drugs was unable to confirm a benefit of hydroxychloroquine or chloroquine, when used alone or with a macrolide, on in-hospital outcomes for COVID-19.^70^ Besides, this study of 96 000 hospitalised patients on six continents found that those who received the drugs had a significantly higher risk of death and an increased frequency of ventricular arrhythmias compared with those who did not use it.^70^

### Treatment of systemic complications in COVID-19

Extracorporeal membrane oxygenation is an excellent choice for patients with ARDS progressing to respiratory failure. Other modes of treatment include high-flow nasal oxygen and endotracheal intubation. Patients experiencing persistent refractory hypoxemia need prone positioning followed by neuromuscular blockade, inhaled nitric oxide (at 5–20ppm) and also provide optimal end-expiratory pressure by inserting oesophagal balloon.^73  74^

In the presence of shock with acute renal failure, negative fluid balance needs to be achieved by dialysis. Antimicrobials are used for pre-exposure and post-exposure prophylaxis. This prevents illness from SARS-CoV-2 and also reduces the risk of acquiring secondary infection. Fluid management is important to reduce pulmonary oedema.^67  68  75^ Glucocorticoids are best avoided due to its harmful effects in viral pneumonia and ARDS.^76^ Rescue therapy by administration of intravenous infusion of vitamin C has been suggested to attenuate vascular injury and systemic inflammation in sepsis and ARDS.^77^

### Role of vaccines

Vaccine development is underway for COVID-19, but there are various limitations. This includes (1) the place for phase 3 vaccine trials are to be conducted in the locality of the ongoing transmission of disease, (2) vaccine manufactures need to work closely with biotechnology companies to develop effective vaccines which probably takes a minimum of 12–18 months and (3) regulators should evaluate safety with a range of virus strains in more than one animal model.^78–80^

The investigational vaccine has been currently developed using mRNA as its genetic platform using prior studies related to SARS and MERS.^16  24^ The basis of effective vaccine is immune targeted and involves identifying of B cell and T cell epitopes derived from the spike (S) and nucleocapsid (N) proteins among 120 available SARS-CoV-2 genetic sequences.^24^ Effective vaccination would play a vital role in reducing the viral spread and eliminate the virus from the host.^81^

## Conclusion

COVID-19 has presented itself as a global pandemic in a short time period resulting in rapid curve shift of infected patients, increasing death rates, huge global economic burden and widespread mobilisation of medical resource across the globe. Being a novel disease, COVID-19 has presented itself as a mystery infection to the medical field, also requiring tremendous research and insights about the nature of the virus, and posing frequent challenges for a successful vaccine outcome. The approach to this disease requires active loco-regional to international collaboration with regards to disease containment, preventive strategies and treatment approach.

**Main messages**

- This article reviews the current state of knowledge concerning the origin, transmission, diagnosis and management of coronavirus disease 2019 (COVID-19).
- It traces the origin of coronavirus as it emerged and differentiates the COVID-19 with specific features from SARS and MERS.
- We give a detailed insight into the modes of transmission, clinical manifestations, diagnosis and management. Also, it highlights the recent trends on vaccine development.

**Key references**

1. Wu F, Zhao S, Yu B, *et al*. A new coronavirus associated with human respiratory disease in China. *Nature* 2020;579:265–9.
2. Raoult D, Zumla A, Locatelli F, *et al*. Coronavirus infections: epidemiological, clinical and immunological features and hypotheses. *Cell Stress* 2020.
3. Chen N, Zhou M, Dong X, *et al*. Epidemiological and clinical characteristics of 99 cases of 2019 novel coronavirus pneumonia in Wuhan, China: a descriptive study. *Lancet* 2020;395:507–13.
4. Dong Y, Mo X, Hu Y, *et al*. Epidemiological characteristics of 2143 pediatric patients with 2019 coronavirus disease in China. *Pediatrics* 2020; e20200702.
5. Cui J, Li F, Shi ZL. Origin and evolution of pathogenic coronaviruses. *Nat Rev Microbiol* 2019;17:181–92.

**Self-assessment questions**

1. Dromedary camel was involved in zoonotic transmission in MERS form of CoV?
2. Based on COVID-19 genomic structure, the protein that mediates an attachment and fusion between the virus and the host cell membrane is membrane (M) protein.
3. Cross-species and human-to-human transmission of COVID-19 is mainly dependent on the host receptor ACE2 protein.
4. Most consistent laboratory finding in COVID-19 patient is lymphopenia
5. COVID-19 is confirmed by RT-PCR.

**Current research questions**

1. How does COVID-19 differ from SARS and MERS?
2. Mention the factors responsible for virus–host interaction.
3. List the specific laboratory findings in suspected cases of COVID-19.
4. Elaborate the scheme of management in COVID-19.

**Self-assessment answers**

- True
- False
- True
- True
- True

## Supplementary Material

postgradmedj-96-753-DC1-inline-supplementary-material-1

postgradmedj-96-753-DC2-inline-supplementary-material-2
