# Supplementary material for: Origin, transmission, diagnosis and management of coronavirus disease 2019 (COVID-19)
Source: Postgrad Med J. 2020 Jun 20;96(1142):753–8. doi: 10.1136/postgradmedj-2020-138234 (PMC10016932; doi:10.1136/postgradmedj-2020-138234)
Supplement: postgradmedj-96-753-DC1-inline-supplementary-material-1 [file postgradmedj-96-753-dc1-inline-supplementary-material-1.pdf]

Title page

Title of the article: Origin, Transmission, Diagnosis and Management of Coronavirus Disease 2019 (COVID-19)

Corresponding author details:

Dr Srikanth Umakanthan
Department of Paraclinical Sciences
Pathology Unit, Building #5, Room 30
Faculty of Medical Sciences
The University of the West Indies
EWMSC, Mount Hope
Email: Srikanth.Umakanthan@sta.uwi.edu

Authors details:

Srikanth Umakanthan, Department of Paraclinical Sciences, Faculty of Medical Sciences, The University of the West Indies, St. Augustine, Trinidad.

Pradeep Kumar Sahu, Department of Centre for Medical Sciences Education, Faculty of Medical Sciences, The University of the West Indies, St. Augustine, Trinidad.

Anu Ranade V, Department of Basic Medical Sciences, University of Sharjah, Sharjah, UAE

Bukelo, Maryann M, Department of Anatomical Pathology, Eric Williams Medical Sciences Complex, North Central Regional Health Authority, Trinidad.

Joseph Sushil R, MD, Department of Surgery, University of Minnesota, Minneapolis, Minnesota, USA

Lucas Faria Abrahao-Machado, Senior Pathologist, Department of Pathology, Botucatu, Sao Paulo, Brazil

Hari Kumar K, Department of International Public Health and Disease Surveillance, Midwest, Australia

Dhananjaya Narayana KV, Department of Radiology, Indragandhi Institute of Health Sciences, Bengaluru, Karnataka, India

Samarika Dahal, Department of Oral Pathology, Institute of Medicine, Maharajgunj, Kathmandu, Nepal

Word counts: text(excluding abstract and references): 2291 words

Source of support: No grants received.

Acknowledgement: NIL

Conflict of Interest: The contributors declare no conflict of interest.
