# Supplementary material for: Origin, transmission, diagnosis and management of coronavirus disease 2019 (COVID-19)
Source: Postgrad Med J. 2020 Jun 20;96(1142):753–8. doi: 10.1136/postgradmedj-2020-138234 (PMC10016932; doi:10.1136/postgradmedj-2020-138234)
Supplement: postgradmedj-96-753-DC2-inline-supplementary-material-2 [file postgradmedj-96-753-dc2-inline-supplementary-material-2.pdf]

The Editor,
BMJ, Postgraduate Medical Journal

18th May, 2020

Sub: Submission of Review article for Publication
Dear Sir,

COVER LETTER

We wish to publish our review article entitled: “Origin, Transmission, Diagnosis and Management of Coronavirus Disease 2019 (COVID-19)” in your esteemed journal. Coronavirus (CoV) has emerged as a global health treat due to its accelerated geographic spread over the last two decades. This article reviews the current state of knowledge concerning the origin, transmission, diagnosis and management of COVID-19.

This manuscript provides a concise and vital link from the disease origin in Wuhan, China to the recent development about vaccine. It also highlights the vital laboratory findings and management of COVID-19 based on various published articles which is very much required for medical professionals to understand.

This article has not been submitted elsewhere.

All the authors have contributed significantly and approved the final version of this manuscript.

We hereby transfer, assign, or otherwise convey all copyright ownership, including any and all rights incidental thereto, exclusively to the journal, in the event that such work is published by the journal.

Thank you very much,

Yours sincerely,

Dr. Srikanth Umakanthan
(Corresponding author)
Department of Paraclinical Sciences,
Faculty of Medical Sciences
The University of the West Indies
St, Augustine, Trinidad and Tobago
Email ID: Srikanth.Umakanthan@sta.uwi.edu

CS Scanned with CamScanner
